# Lost Polarization of Aquaporin4 and Dystroglycan in the Core Lesion after Traumatic Brain Injury Suggests Functional Divergence in Evolution 

**DOI:** 10.1155/2015/471631

**Published:** 2015-10-25

**Authors:** Hui Liu, Gou ping Qiu, Fei Zhuo, Wei hua Yu, Shan quan Sun, Fen hong Li, Mei Yang

**Affiliations:** ^1^Institute of Neuroscience, Chongqing Medical University, Chongqing 400016, China; ^2^Department of Mathematics and Computation Science, Shangluo University, Shangluo, Shanxi 726000, China

## Abstract

*Objective*. To understand how aquaporin4 (AQP4) and dystroglycan (DG) polarized distribution change and their roles in brain edema formation after traumatic brain injury (TBI). *Methods*. Brain water content, Evans blue detection, real-time PCR, western blot, and immunofluorescence were used. *Results*. At an early stage of TBI, AQP4 and DG maintained vessel-like pattern in perivascular endfeet; M1, M23, and M1/M23 were increased in the core lesion. At a later stage of TBI, DG expression was lost in perivascular area, accompanied with similar but delayed change of AQP4 expression; expression of M1, M23, and DG and the ratio of M1/M2 were increased. *Conclusion*. At an early stage, AQP4 and DG maintained the polarized distribution. Upregulated M1 and M23 could retard the cytotoxic edema formation. At a later stage AQP4 and DG polarized expression were lost from perivascular endfeet and induced the worst cytotoxic brain edema. The alteration of DG expression could regulate that of AQP4 expression after TBI.

## 1. Introduction

Brain edema is a common consequence of traumatic brain injury (TBI). Two main types of edema are distinguished. Cytotoxic edema is caused by energy failure and subsequent accumulation of fluid in the cell resulting in cell swelling, while vasogenic edema occurs when the blood-brain barrier (BBB) becomes leaky permitting influx of plasma constituents into the brain extracellular space [1]. The cellular and molecular mechanisms contributing to the development/resolution of TBI-associated brain edema are poorly understood. Recent data suggest that aquaporin (AQP) water channels may have a central role in keeping water homeostasis in the brain [2]. AQP4, the primary water channel in brain, is expressed on ependymal cells and astrocytes. Astrocytes are highly branched cells with processes that contact most of the surfaces of neuronal dendrites and cell bodies as well as some axonal surfaces and synapses. Other astrocyte processes that end in expansions are called endfeet [3]. Tenfold higher density of AQP4 exhibit on endfoot membranes than nonendfoot membranes is called the polarized expression of AQP4.

Actually, AQP4 in endfeet membrane forms large orthogonal arrays of particles (OAPs) [4, 5]. Normally AQP4 forms tetramer. The tetramers then form OAPs, which consist of four to more than one hundred tetramers [6, 7]. Two isoforms of AQP4 are identified, M1 and M23. M23 exhibits a much greater water transport capacity than M1 and it usually forms stable and large raft-like lattices of OAPs, whereas M1 forms no or very small arrays [5]. Recently, studies suggest that the polarized distribution of AQP4 is associated with dystrophin-dystroglycan complex (DDC) [8]. The central component of the DDC is dystroglycan (DG), which comprises *α*- and *β*-subunits. *α*-DG binds to extracellular matrix (ECM) components such as agrin and laminin, whereas *β*-DG is a transmembrane protein connecting *α*-DG with the cytoskeleton and other intracellular components such as *α*-syntrophin, which provides a combining site for AQP4 [5].

Study in DG-knockout mouse revealed a selective loss of AQP4 in perivascular astroglial endfeet with decreased number of OAPs but no significant change of AQP4 expression on superficial astroglial endfeet with no OAPs formation [5]. Research on cultured astrocytes showed that DG silencing reduced the number and area of laminin induced clusters of AQP4 and provided evidence of DDC regulating AQP4 polarization in astrocytes [9]. Another study in C57BL/6 mice after 30 min transient middle cerebral artery occlusion demonstrated that in both the core lesion and the penumbra *β*-DG is lost from astrocyte endfeet, accompanied by a loss of polarized localization of AQP4 without developing early cytotoxic edema [1]. However, so far little research has been done about the change of AQP4 polarization and the relation between AQP4 and DG in vivo after TBI, which affects twice as many patients as stroke injury [10]. To understand it, this study was carried out.

## 2. Animal and Methods

### 2.1. Experimental Animal, Groups, and Tissue Collection

The study was in accordance with the Guide for the Care and Use of Laboratory Animals of NIH. 368 male Sprague Dawley rats, weighing 250 ± 50 g, supplied by the Experimental Animal Centre of Chongqing Medical University, were randomly divided into operation and sham-operation groups (as control). Four, 4, 3, 6, and 6 rats were used, respectively, for real-time PCR, western blot (WB), immunofluorescence (IF), brain water content (BWC), and Evans blue (EB) detection at each time point of each group. The core lesion of brain cortex was isolated for quantitative PCR, WB, BWC, and EB detection and whole brain was removed for IF at 1, 3, 6, 12, 24, 48, and 72 h and 1 week.

### 2.2. Model Establishment

According to the previous methods [11, 12], TBI model was established. The animals were deeply anesthetized with ketamine (80 mg/kg i.p.) and fixed in a stereotaxic frame. The skull was exposed, and the dura was kept intact after the craniectomy in the right parietal region between coronary and lambdoid sutures in a diameter of 5 mm. The experimental severe TBI was performed by dropping 40 g metal weight through a stainless steel guide staff (25 cm) on a brass foot plate placed over the dura. The sham-operation group suffered the same operation except the weight dropped on the foot plate.

After the operation, balance beam was used to evaluate the neurobehavioral deficits [13, 14]. A beam of wood 90 cm long, 1.5 cm wide, and 1.5 cm thick, suspended 45 cm off the ground with an open platform at the start end and a partially enclosed (three walls) platform containing a food reward at the finish end, was used to obtain the score of the animal's ability of maintaining balance. Scores were averaged across three trials per observation. Categorical score data (1–4 possible) is as follows: (1) unimpaired (rat walked smoothly all the way across with well-placed feet and no hesitation or upset balance); (2) slightly impaired (rat walked all the way across mostly smoothly with some hesitation and slips); (3) moderately impaired (rat dragged feet or missed the beam when walking, slipped often, may have fallen, and may not have completed the full distance); (4) severely impaired (rat slipped with most steps, fell at least once, was hesitant, and may have been unable to traverse half the distance). Sham-operation rat with “unimpaired” score was chosen as control group. Operated rat with “severely impaired” score was chosen as TBI group.

### 2.3. Brain Water Content

Brain cortices were weighed (wet weight) and dried in vacuum oven at 120°C for 48 h. The dried brain was reweighted (dry weight). The percent of water content was calculated as ([wet weight − dry weight]/wet weight) × 100%.

### 2.4. Evans Blue (EB) Detection

EB (2% in saline; 2 mL/kg) was intravenously administered by internal carotid vein 1 h before rats were sacrificed. The rats were perfused transcardially with 0.01 M PBS and the brain cortices were removed, weighed, and homogenized. 4 mL 99.5% formamide per gram of tissue was added and placed on a shaker for 48 hours. Supernatants were collected and measured with a spectrophotometer at 620 nm and compared against a standard curve. The results were expressed as micrograms of albumin-EB/milligram of brain tissue.

### 2.5. Quantitative Real-Time PCR Analysis

Total RNA was extracted using Tissue/cell RNA Mini kit (Invitrogen) according to the manufacturer's protocol. The cDNAs were generated from 1 *μ*g of total RNA by superscript II RNase H-reverse transcriptase with Oligo (dT) primer. Quantitative PCR reaction was achieved using commercial kit containing SYBR Green (Eurogentec, Seraing, Belgium). The primer pairs (Invitrogen) were as follows: AQP4 (MN-012825, 5′-agaaccaaggcgtaaaccg-3′ and 5′-tccctggaaatgactgagaaa-3′, 256 bp), DG (NM_053697.1, 5′-tagcgtccctgacatccg-3′ and 5′-gaatcagttgaaggcgttgc-3′, 516 bp), and *β*-actin (NM_031144, 5′-ctgccgcatcctcttcctc-3′ and 5′-ctcctgcttgctgatccacat-3′, 398 bp). The cycling program was 5 min at 95°C and then 40 cycles, each consisting of 15 s at 95°C and 30 s at 60°C. Changes in each mRNA expression were examined with ABI7000 Sequence Detection System (Perkin Elmer, USA). A standard curve was used to extrapolate the copy number of target cDNA in rat brain.

### 2.6. Immunohistochemistry

The frozen serial coronal sections (10 *μ*m) were sliced and blocked in 0.01 M PBS containing 10% horse serum for 6 h at 4°C. AQP4 rabbit monoclonal (1 : 200, Abcam), *α*-DG goat polyclonal (1 : 200, Santa Cruz), and *β*-DG mouse monoclonal antibody (1 : 200, Abcam) were used. Sections were incubated in the mixed primary antibody overnight at 4°C and then with Cy5-labeled goat anti-rabbit IgG (1 : 200, Chemicon) plus TRITC-labeled rabbit anti-goat IgG (1 : 200, Santa Cruz) and FITC-labeled goat anti-mouse IgG (1 : 200, Santa Cruz) overnight at 4°C. Finally, sections were stained with DAPI (Beyorime) and then mounted and imaged in a confocal scanning microscope (Leica TCS SP2, Wetzlar, Germany).

### 2.7. Western Blot

The brain cortices were homogenized in ice-cold lysis buffer (Beyotime) with PMSF (final concentration 1 mM) for 15 min and centrifuged at 12,000 rpm at 4°C for 10 min. The supernatants were collected and boiled with 5x sample buffer at 95°C for 5 min. Then the samples were separated by SDS/PAGE and transferred onto PVDF membrane at 350 mA for 55 min at 4°C. The membranes were blocked with 10% horse serum in 0.01 M PBS for 2 h, probed with AQP4 (1 : 200, rabbit polyclonal antibody, Chemicon), *α*-DG and *β*-DG (1 : 200), and *β*-actin (1 : 5000, mouse monoclonal antibody, Santa Cruz) antibody overnight at 4°C. The blots were incubated with horse radish peroxidase conjugated antibody (1 : 10000 for *β*-actin, 1 : 500 for other antibodies, Santa Cruz) for 4 h. The specific reaction was visualized by using a chemiluminescent substrate (Pierce, USA). The bands were quantified by gel densitometry (Bio-Rad, Hercules, USA). The value of individual protein band was divided by the value of *β*-actin from the same sample; a ratio of protein/*β*-actin for each sample was obtained.

### 2.8. Statistical Analysis

All statistics were performed using the SPSS 11.0 software package (Chicago, IL, USA). The data were analyzed by two-tailed *t*-tests or independent test.

## 3. Results

### 3.1. Brain Water Content

**Figure 1:**
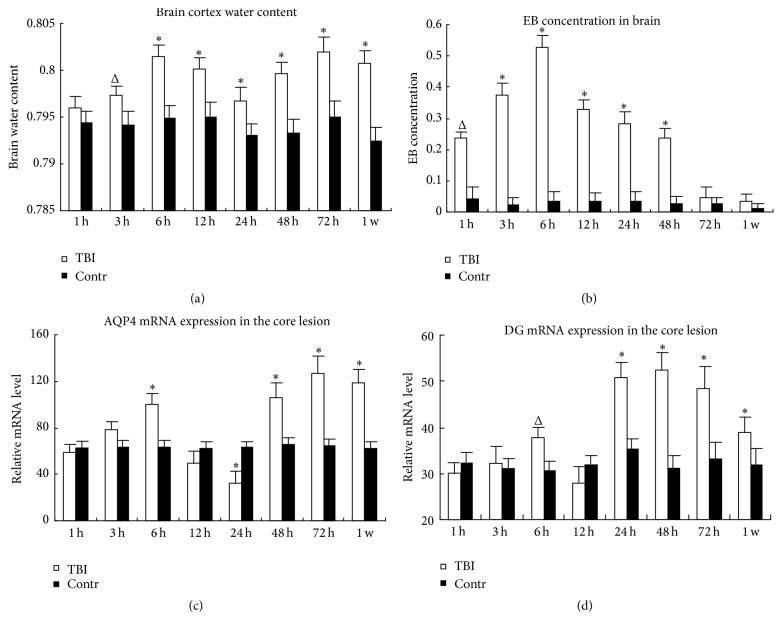
The changes of brain water content (a), BBB permeability (b), AQP4 (c), and DG (d) mRNA expression in the core lesion of brain cortex after TBI. TBI: traumatic brain injury group. Contr: the sham-operation group. ^Δ^ *P* < 0.05. ^*∗*^ *P* < 0.01.

To reveal the degree of brain edema, brain water content was detected. Compared with control group, water content was increased obviously and reached the first peak at 6 h (*P* < 0.01). Following a slight decrease at 12 and 24 h, which was still higher than control (*P* < 0.01), it increased and reached the highest peak at 72 h (*P* < 0.01). Until 1 week, it was still higher than control group (Figure 1(a)). It showed two peaks of brain edema formed after TBI. The worst one occurred at 72 h after TBI.

### 3.2. Evans Blue Concentration

To detect the disruption of BBB and to address the course of vasogenic brain edema formation after TBI, EB was intravenously injected and the concentration in brain was detected. EB concentration was increased rapidly after 1 h and reached the peak at 6 h and then slowly decreased and there was no obvious difference after 72 h in the core lesion of brain cortex (Figure 1(b)). It suggested the BBB was impaired severely before 72 h, and the worst damage happened at 6 h after TBI.

### 3.3. Real-Time PCR

AQP4 and DG mRNA were both increased at 6 h and then downregulated at 12 h. AQP4 mRNA continued to decrease (*P* < 0.01), while DG mRNA was already increased at 24 h. After that, they were increased and reached the peak at 72 h and 48 h, respectively. Although AQP4 mRNA changed one step behind that of DG, the changing trend of AQP4 and DG mRNA was almost accordant and shows temporal correlation (Figures 1(c) and 1(d)).

### 3.4. Western Blot

**Figure 2:**
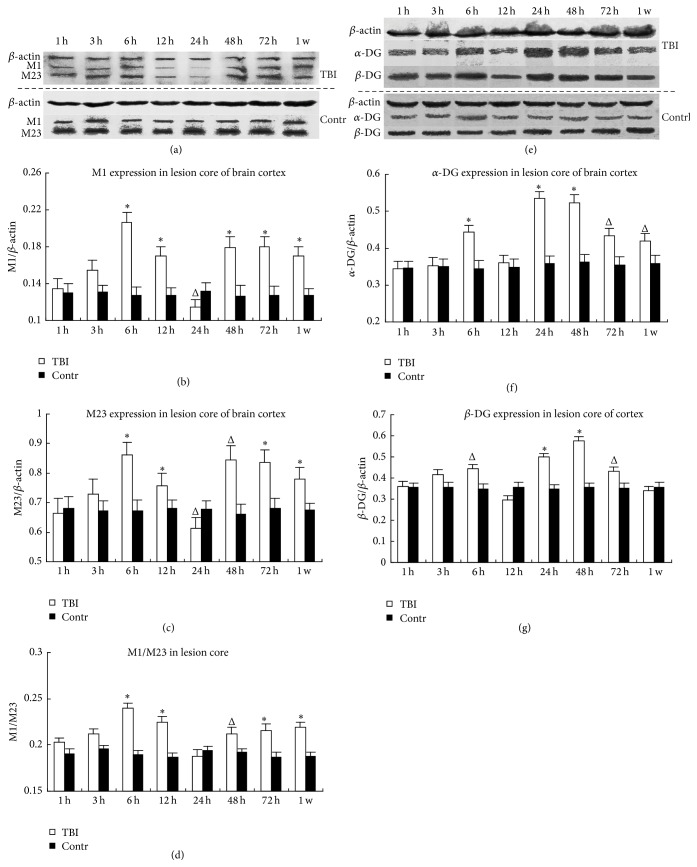
The abundance of AQP4, *α*-DG, and *β*-DG in the core lesion of brain cortex in WB. The left and right column, respectively, showed the expression of AQP4 (a–d) and DG (e–g) proteins in the lesion core of TBI group and the same area of control group. The picture (a, e) above and under the dash line represented TBI and control group, respectively. The molecular weight of *β*-actin, AQP4-M1, AQP4-M23, GAPDH, *α*-DG, and *β*-DG was 43, 34, 28–30, 37, 120, and 43 kD. TBI: traumatic brain injury group; Contr: the sham-operation group. ^Δ^ *P* < 0.05. ^*∗*^ *P* < 0.01.

*α*-DG and *β*-DG were obviously increased at 6 h, followed a slight decrease at 12 h (*α*-DG decreased more than *β*-DG); then they increased again and reach the highest peak at 24 or 48 h. AQP4 M1 and M23 isoforms were increased at 6 h and then obviously downregulated at 24 h; then they increased again at 48 h or 72 h. The changes of AQP4 and DG were almost accordant. Additionally, the ratio of AQP4 M1 to M23 was increased almost in every time point, except 24 h (Figure 2).

### 3.5. Immunofluorescence

**Figure 3:**
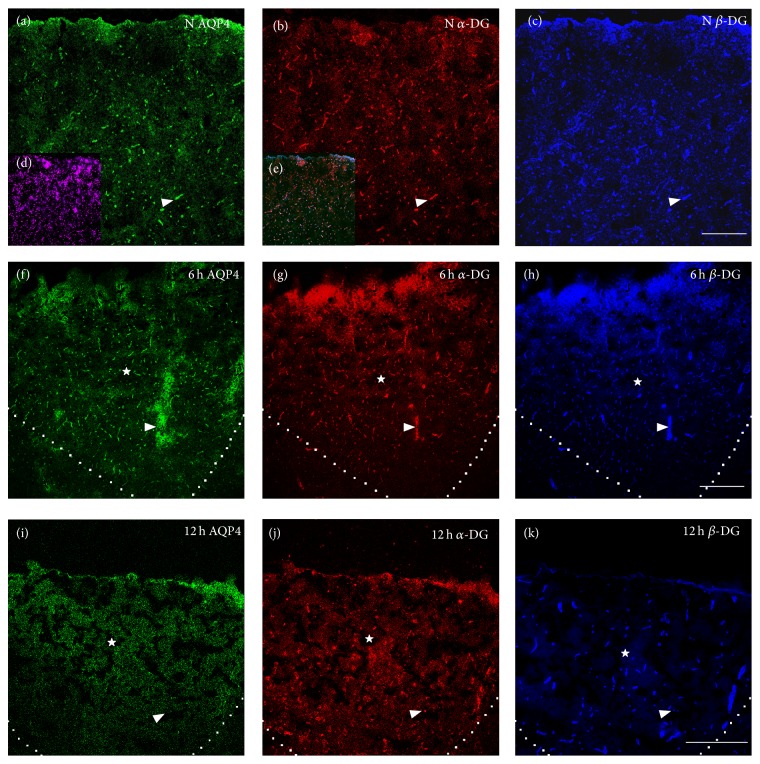
The expression of AQP4, *α*-DG, and *β*-DG in normal brain cortex and in the core lesion at 6 and 12 h after TBI. Green, red, and blue IF signal represented AQP4, *α*-DG, and *β*-DG, respectively. Small panels (d) and (e) showed DAPI staining and the merged images, respectively. The area surrounded by dashed line or indicated by star (☆) was the core lesion. Normally, AQP4, *α*-DG, and *β*-DG were specially abundant in perivascular endfeet of astrocyte and vessel-like sharp appeared indicated by arrow (△) in (a)–(c). At 6 h after TBI, the vessel-like sharp was maintained and the IF signals of the three proteins were increased in the core lesion shown by (f)–(h). At 12 h, the three proteins were dramatically decreased in perivascular endfeet and punctation-like sharp appeared indicated by arrow (△) and AQP4 and *α*-DG were lost more seriously than *β*-DG in (i)–(k). Scale bar: 150 *μ*m.

**Figure 4:**
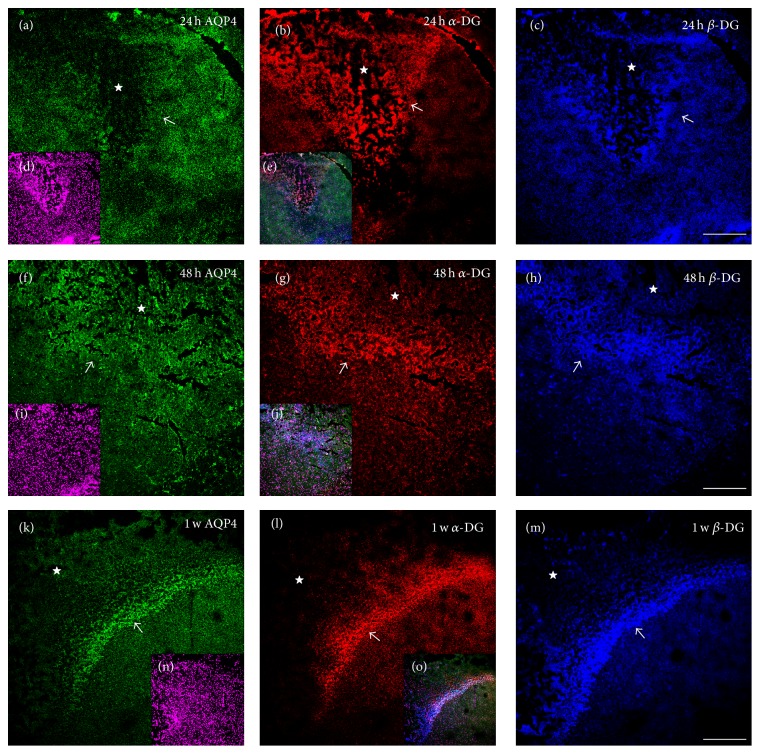
The changes of AQP4, *α*-DG, and *β*-DG expression in the penumbra and core lesion of brain cortex after 24 h of TBI. Green, red, and blue IF signal represented AQP4, *α*-DG, and *β*-DG, respectively. Small panels (d), (i), and (n) showed DAPI staining. Small panels (e), (j), and (o) were the merged images. The area surrounded by dashed line or indicated by star (☆) was the core lesion. Arrow (↗) indicated the penumbra of the core lesion. At 24 h, the polarized expression of three proteins in perivascular endfeet was totally lost in the lesion core and the surrounding core area, although *α*-DG and *β*-DG were diffusedly increased, markedly, in the lesion core shown by (a)–(c). At 48 h, an obvious penumbra surrounding the lesion core indicated by arrow in (f)–(h) appeared, although AQP4 IF signal was weak but visible. Contrasted with 24 h, at 48 h shown in (f)–(h), the three signals are still lost or diminished in perivascular area. The diffused expression of *α*-DG was decreased in the lesion core but greatly increased in the penumbra; *β*-DG continues to be increased in the lesion core and the penumbra; AQP4 was dramatically and diffusedly increased in the lesion core and penumbra (weakly but visible). Until 1 w, the three signals were greatly increased in penumbra but decreased slightly in the lesion core shown in (k)–(m). Scale bar: 150 *μ*m.

To detect the change of the distribution and colocalization of AQP4 and DG expression, immunostaining was used. Before 6 h after TBI, no obvious change was found in distribution of AQP4, *α*-DG, and *β*-DG IF staining patterns, in which the IF signals were still strong in the perivascular endfeet and a vessel-like sharp appeared (Figure 3(a–h), indicated in arrowhead) in the core lesion. However, at 12 h, the three IF signals were sharply decreased in the core lesion and presented with a punctate pattern (Figure 3(i–k), indicated in arrowhead) rather than the original vessel-like sharp, although *β*-DG IF signal decreased less than the other two. At 24 h, although diffused AQP4 signal could be detected in surrounding core area, the vessel-like expression was almost lost. Moreover, AQP4 IF signal was almost absent in the core lesion. Meanwhile *α*-DG and *β*-DG IF signal were also lost from perivascular area in the core lesion; however, they enhanced dramatically and diffusedly in the core lesion and specially in penumbra (Figure 4(a–e)). At 48 h, diffused and slightly increased AQP4 IF signal was visible again in core lesion and penumbra, whereas DG IF signals were still increased diffusedly in the core lesion and strongly increased in penumbra (Figure 4(p–o)). Although AQP4 IF signal was weaker than DG in the core lesion and penumbra at 48 h, it increased gradually and has the similar fluorescence intensity with DG in the core lesion and penumbra after 72 h.

In conclusion, after TBI, DG IF signals were lost from perivascular area in the core lesion after 6 h and then increased diffusedly in the core lesion and then in penumbra. Although AQP4 was lost synchronously with *α*-DG at 12 h, the change of AQP4 was one step behind that of DG: DG was diffusedly increased in the core lesion at 24 h, while AQP4 increased at 48 h; DG was dramatically increased in penumbra at 48 h, while AQP4 only slight increased at 48 h and dramatically increased at 72 h. These results strongly indicated that the polarization of AQP4 and DG was lost and the change of AQP4 expression was closely associated with that of DG after TBI.

## 4. Discussion

### 4.1. AQP Family and AQP4

The main mechanism for water recruitment was believed by passive diffusion 30 years ago. Until the 1990s, the identification of the first membrane intrinsic proteins (MIPs) in human not only changed dramatically the mainstream view but also prompted the search for homologues [15]. MIPs, an ancient, ubiquitous, highly diversified, and life essential protein family, also known as AQPs, are channel proteins which facilitate the transport of water and small solutes, such as glycerol, urea, ammonia, metalloids, and carbon dioxide, across cell membranes in all living organisms [16, 17]. The great diversity of forms and functions displayed by the different members of the MIP family can only be fully understood within an evolutionary framework [16]. Based mainly on sequence similarity, evolutionary relationships among the members (i.e., paralogues) of MIP family are classified into six major paralogous groups: (1) GLPs or glycerol-transporting channel proteins; (2) AQPs, which include metazoan AQP0, 1, 2, 4, 5, and 6; (3) PIPs or plasma membrane intrinsic proteins of plants; (4) TIPs or tonoplast intrinsic proteins of plants; (5) NODs or nodulins of plants; and (6) AQP8s [17]. There is also the earliest simple way from a phylogenetic perspective, according to the function, classifying the MIP family into water channels and glycerol facilitators [16]. Animal AQPs can be classified into three major groups. The first group includes classical AQP4, 1, 0, 5, 6, and 2, which are found in vertebrates, and are likely the result of whole genome duplications at early stages of their evolutionary history. The second group includes AQP8 orthologs found from nematodes to mammals. The third group includes AQP11 and AQP12, which are only found in ray-finned fishes and sarcopterygians [16]. The most basal ones in the first group, AQP1 and AQP4 are distributed widely in kidney, lungs, brain, stomach, eye, and ear in human [15] and AQP4 is clearly the most expressed paralog in brain [16].

AQPs contain six membrane-spanning segments with five connecting loops (A–E). The N- and C-terminal halves of AQPs share about 20% sequence identity. This genetic relation suggests that the AQP family arose by tandem gene duplication during evolution. The cytoplasmic loop B and the extracellular loop E, containing a tripeptide signature motif asparagine-proline-alanine (NPA), are structural components of the aqueous pore since they are hydrophobic in nature [18].

AQP4, a small 30 kDa monomer, also has these domains. The molecule spans the cell membrane 6 times, forming 5 interhelical loops designated as A, C, and E on the extracellular surface and B and D on the intracellular surface. NPA motifs are present in both B and E loops. Each monomer folds into a structure that forms an independent water channel, characterized by wide external openings and a narrow central constriction where the NPA motifs interact. AQP4 monomers assemble into tetramers, with each monomer being individually functional. Water movement through the channel is governed by an osmotic gradient across the membrane, with flow limited by size restriction and electrostatic repulsion [19].

### 4.2. AQP4 and Brain Edema after TBI

Although several studies have been done about AQP4 expression in brain after TBI, the results were inconsistent. Some experiments showed that AQP4 expression was increased in the core lesion of the patients suffering TBI [20] and in the peri-injury area at 24 and 72 h in rats after penetrating brain injury [21]. Some research showed that AQP4 expression changed differently in different area. Sun et al. [17] found that AQP4 mRNA was increased at the injury site and decreased adjacent to the injury site, and there was no difference at a site distant from the injury in rat at 24 h after TBI. However, Ren et al. [22] found that global AQP4 expression was generally increased, but AQP4 lost its polarized localization at endfoot processes of reactive astrocytes in mice after TBI. Additionally, some studies showed AQP4 expression was decreased in the cerebral cortex at 24 h compared with 12 h in mice [23] and in both hemispheres in rats after TBI [24]. The different results may be caused by the difference of the study subjects, the area detected, the measure used to establish TBI model, and the degree of the TBI. In our study, we focused on the core lesion and the penumbra and found that AQP4 and its mRNA was increased almost at all the time points after TBI, except 24 h, and AQP4 was lost in perivascular area after 12 h.

According to the pathophysiological change, TBI can be divided into primary processes, usually at an early stage with vasogenic edema, and secondary processes, usually at a later stage with cytotoxic brain edema. According to the data of our study, the early stage referred to the period within 6 h, which is characterized with the severe BBB disruption and AQP4 was still clustered in the perivascular endfeet. After TBI, the fluid in blood flowed into the intercellular space through the disrupted BBB and induced vasogenic edema at an early stage in our study. At the same time, AQP4 still maintained vessel-like expression in the core lesion, suggesting that the polarized distribution of AQP4 was maintained. However, M1 and M23 and the ratio of M1 to M23 were all increased in the core lesion at this stage. Study indicated that M1 usually formed no or small number of OAPs and is abundant in the nonendfoot membrane with low capability of water permeation, whereas M23 usually forms large number of OAPs and is abundant in the endfeet membrane with high capability of water permeation [5]. It suggested that more small number of OAPs or just AQP4 tetramers were formed in nonendfeet or other endfeet membranes with the lower capability of water permeation. Although the vessel-like sharp is still maintained, the polarized AQP4 distribution was destroyed partly. Similar results that were found in studies of ischemia where M1 was increased at an early stage were correlated with reduced edema and less water in the tissue [25]. Combining our results, the worst BBB disruption at 6 h is accompanied with the increased M1 and M23 expression and the ratio of M1 to M23 but not with the worst brain edema. It suggested that upregulated M1 in endfeet or nonendfoot membranes with the lower water permeation could slow down the water flowing from intercellular space into astrocytes and retarded the cytotoxic brain edema formation. Meanwhile, the increased M23, which is still abundant in perivascular endfeet, could help to remove the excess liquid into blood vessel and to extenuate the brain edema in the core lesion.

At a later stage of TBI, the loss of AQP4 in perivascular endfeet and the diffusedly increased AQP4 in the core lesion demonstrated that the polarized distribution of AQP4 was lost and redistributed in other endfeet or nonendfoot membrane of astrocytes. The increased ratio of M1 to M23 in the core lesion also suggested that the polarized distribution of AQP4 was lost. Overall, the loss of AQP4 from perivascular endfeet could induce the disorder of clearing the excess water to vessels; meanwhile the diffusedly increased AQP4 in other endfeet or nonendfeet membrane could facilitate the water moving into the astrocytes and finally induce the worst cytotoxic brain edema at a later stage of TBI.

### 4.3. Alteration of DG Could Regulate the Change of AQP4

Via adhesion receptors, integrins, and DG, astrocytes and microvessel endothelium were anchored to the ECM [26, 27] and the adhesion via DG contributed to regulation of water transport by astrocytes [27]. The disruption of DG-laminin interaction impaired the ability of astrocytes to direct water transport [27] and acute loss of DG could diminish the ability of astrocytes to resolve edema [28]. Astrocytes regulated the brain fluid balance between the extracellular and intracellular space via Kir 4.1 and AQP4 [27], which can be regulated by DG [29]. We found that DG and AQP4, maintaining their polarized distribution, were both increased at the early stage. However, at the later stage, although the changing trend of AQP4 and DG was accordant, AQP4 expression changed one step behind that of DG in both the core lesion and the penumbra. Our results suggested that DG could regulate the expression of AQP4 after TBI. The increased DG in the early stage upregulated AQP4 expression in perivascular and subpial endfeet which could help to remove the water accumulated in the extracellular space to vascular or subarachnoid space. The loss of the polarized distribution of DG induced the same change of AQP4 and thus leaded to the worst cytotoxic brain edema at the later stage of TBI. Why could the alteration of DG regulate the expression of AQP4? Recent research provided some clues.

Astrocytes rapidly swelled in response to OGD, hyposmotic stress, elevated extracellular K^+^, oxidative stress, or traumatic injury in vitro via activation of extracellular signal-related kinase (ERK) [27]. DG was a signaling scaffold for ERK [30] and was a mechanosensitive transducer of cell stretching via an ERK-dependent mechanism in lung alveolar cells [31] and astrocytes [32]. It also could initiate signaling that mediated compensatory responses such as upregulation of channel expression and/or alteration of their cellular distribution [32]. ERK activation was dependent on DG in ischemic astrocytes [27]. After TBI, the stretch on astrocytes caused by the accumulated fluid in extracellular space came from blood vessel, was captured by DG, as a mechanosensitive transducer of cell stretching and activated ERK pathway, and finally induced the up regulation of AQP4 in the perivascular and subpial endfeet at 6 h after TBI. However, at the same time, BBB was disrupted seriously, which was usually coupled with the inflammatory response and activation of matrix metalloproteinases (MMP) [33]. The Increased MMP-2, 9 expression led to loss of DG, which is involved in the disruption of the regular assembly and expression of AQP4 [34, 35]. Thus *α*-DG and *β*-DG were decreased progressively after 6 h and were finally lost after 24 h from perivascular endfeet, followed by the similar but “one step behind” change of AQP4.

Previous study showed that cultured astrocytes, suffering from traumatic injury, became “reactive.” They hypertrophied and polarized dramatically and secreted ECM proteins and then extended processes towards the region of the wound, forming a “scar” and also secreted ECM proteins that isolated the wound [36]. Responding to the increased ECM secreted by activated astrocytes, *α*-DG and *β*-DG were upregulated again and diffusedly expressed in the lesion core at 24 h and then were dramatically increased in penumbra after 48 h to isolate the wound, followed by the postponed expression of AQP4 regulated by DG via activated ERK pathway.

## References

[1] Steiner E., Enzmann G. U., Lin S. (2012). Loss of astrocyte polarization upon transient focal brain ischemia as a possible mechanism to counteract early edema formation. *Glia*.

[2] Yang M., Gao F., Liu H. (2011). Immunolocalization of aquaporins in rat brain. *Anatomia, Histologia, Embryologia*.

[3] Nakazawa E., Ishikawa H. (1998). Ultrastructural observations of astrocyte end-feet in the rat central nervous system. *Journal of Neurocytology*.

[4] Fallier-Becker P., Sperveslage J., Wolburg H., Noell S. (2011). The impact of agrin on the formation of orthogonal arrays of particles in cultured astrocytes from wild-type and agrin-null mice. *Brain Research*.

[5] Noell S., Wolburg-Buchholz K., Mack A. F. (2011). Evidence for a role of dystroglycan regulating the membrane architecture of astroglial endfeet. *The European Journal of Neuroscience*.

[6] Rash J. E. (2010). Molecular disruptions of the panglial syncytium block potassium siphoning and axonal saltatory conduction: pertinence to neuromyelitis optica and other demyelinating diseases of the central nervous system. *Neuroscience*.

[7] Wolburg H., Noell S., Fallier-Becker P., MacK A. F., Wolburg-Buchholz K. (2012). The disturbed blood-brain barrier in human glioblastoma. *Molecular Aspects of Medicine*.

[8] Amiry-Moghaddam M., Frydenlund D. S., Ottersen O. P. (2004). Anchoring of aquaporin-4 in brain: molecular mechanisms and implications for the physiology and pathophysiology of water transport. *Neuroscience*.

[9] Noël G., Tham D. K. L., Moukhles H. (2009). Interdependence of laminin-mediated clustering of lipid rafts and the dystrophin complex in astrocytes. *The Journal of Biological Chemistry*.

[10] Laird M. D., Vender J. R., Dhandapani K. M. (2008). Opposing roles for reactive astrocytes following traumatic brain injury. *NeuroSignals*.

[11] Feeney D. M., Boyeson M. G., Linn R. T., Murray H. M., Dail W. G. (1981). Responses to cortical injury: I. Methodology and local effects of contusions in the rat. *Brain Research*.

[12] Liu H., Yang M., Qiu G.-P. (2012). Aquaporin 9 in rat brain after severe traumatic brain injury. *Arquivos de Neuro-Psiquiatria*.

[13] Thullier F., Lalonde R., Cousin X., Lestienne F. (1997). Neurobehavioral evaluation of lurcher mutant mice during ontogeny. *Developmental Brain Research*.

[14] Witholt R., Gwiazda R. H., Smith D. R. (2000). The neurobehavioral effects of subchronic manganese exposure in the presence and absence of pre-parkinsonism. *Neurotoxicology and Teratology*.

[15] Zardoya R. (2005). Phylogeny and evolution of the major intrinsic protein family. *Biology of the Cell*.

[16] Abascal F., Irisarri I., Zardoya R. (2014). Diversity and evolution of membrane intrinsic proteins. *Biochimica et Biophysica Acta*.

[17] Sun M.-C., Honey C. R., Berk C., Wong N. L. M., Tsui J. K. C. (2003). Regulation of aquaporin-4 in a traumatic brain injury model in rats. *Journal of Neurosurgery*.

[18] Jung J. S., Prestont G. M., Smith B. L., Guggino W. B., Agre P. (1994). Molecular structure of the water channel through aquaporin CHIP: the hourglass model. *The Journal of Biological Chemistry*.

[19] Verkman A. S. (2005). More than just water channels: unexpected cellular roles of aquaporins. *Journal of Cell Science*.

[20] Hu H., Yao H.-T., Zhang W.-P. (2005). Increased expression of aquaporin-4 in human traumatic brain injury and brain tumors. *Journal of Zhejiang University: Science B*.

[21] Neal C. J., Lee E. Y., Gyorgy A., Ecklund J. M., Agoston D. V., Ling G. S. F. (2007). Effect of penetrating brain injury on aquaporin-4 expression using a rat model. *Journal of Neurotrauma*.

[22] Ren Z., Iliff J. J., Yang L. (2013). ‘Hit & Run’ model of closed-skull traumatic brain injury (TBI) reveals complex patterns of post-traumatic AQP4 dysregulation. *Journal of Cerebral Blood Flow & Metabolism*.

[23] Kimbler D. E., Shields J., Yanasak N., Vender J. R., Dhandapani K. M. (2012). Activation of P2X7 promotes cerebral edema and neurological injury after traumatic brain injury in mice. *PLoS ONE*.

[24] Kiening K. L., van Landeghem F. K. H., Schreiber S. (2002). Decreased hemispheric Aquaporin-4 is linked to evolving brain edema following controlled cortical impact injury in rats. *Neuroscience Letters*.

[25] Fu D., Lu M. (2007). The structural basis of water permeation and proton exclusion in aquaporins. *Molecular Membrane Biology*.

[26] Baeten K. M., Akassoglou K. (2011). Extracellular matrix and matrix receptors in blood-brain barrier formation and stroke. *Developmental Neurobiology*.

[27] Hawkins B. T., Gu Y.-H., Izawa Y., del Zoppo G. J. (2013). Disruption of dystroglycan-laminin interactions modulates water uptake by astrocytes. *Brain Research*.

[28] Papadopoulos M. C., Manley G. T., Krishna S., Verkman A. S. (2004). Aquaporin-4 facilitates reabsorption of excess fluid in vasogenic brain edema. *The FASEB Journal*.

[29] Qi L.-L., Fang S.-H., Shi W.-Z. (2011). CysLT2 receptor-mediated AQP4 up-regulation is involved in ischemic-like injury through activation of ERK and p38 MAPK in rat astrocytes. *Life Sciences*.

[30] Spence H. J., Dhillon A. S., James M., Winder S. J. (2004). Dystroglycan, a scaffold for the ERK-MAP kinase cascade. *EMBO Reports*.

[31] Jones J. C. R., Lane K., Hopkinson S. B. (2005). Laminin-6 assembles into multimolecular fibrillar complexes with perlecan and participates in mechanical-signal transduction via a dystroglycan-dependent, integrin-independent mechanism. *Journal of Cell Science*.

[32] Cai L., Du T., Song D., Li B., Hertz L., Peng L. (2011). Astrocyte ERK phosphorylation precedes K^+^-induced swelling but follows hypotonicity-induced swelling. *Neuropathology*.

[33] Berezowski V., Fukuda A. M., Cecchelli R., Badaut J. (2012). Endothelial cells and astrocytes: a concerto en duo in ischemic pathophysiology. *International Journal of Cell Biology*.

[34] Noell S., Wolburg-Buchholz K., Mack A. F. (2012). Dynamics of expression patterns of AQP4, dystroglycan, agrin and matrix metalloproteinases in human glioblastoma. *Cell and Tissue Research*.

[35] Zhao W.-J., Zhang W., Li G.-L., Cui Y., Shi Z.-F., Yuan F. (2012). Differential expression of MMP-9 and AQP4 in human glioma samples. *Folia Neuropathologica*.

[36] Peng H., Carbonetto S. (2012). Astrocyte polarization and wound healing in culture: studying cell adhesion molecules. *Methods in Molecular Biology*.

